# Fission-track data and U–Pb dating of granites from Cameron Highland, Peninsular Malaysia: Evidence to comprehend exhumation episodes

**DOI:** 10.1016/j.dib.2019.104162

**Published:** 2019-06-21

**Authors:** Solomon Kassa, Haylay Tsegab, Chow Weng Sum, Choong CheeMeng

**Affiliations:** a Department of Applied Geology, School of Applied Natural Science, Adama Science and Technology University, Adama, Ethiopia; b Department of Geosciences, Faculty of Geosciences and Petroleum Engineering, Universiti Teknologi PETRONAS, 32610, Bandar Sngineeringeri Iskandar, Perak, Malaysia

**Keywords:** Cameron highland, Fission-track, Main range granite, Thermal modeling

## Abstract

Fission tracks are linear trails of intense radiation damage in the crystal structure of a mineral, produced by spontaneous fissioning of uranium-238 (^238^U) atoms. Detail information on the low-temperature thermal histories of rocks, below∼120 °C for tracks in apatite and below∼350 °C for zircon, can be provided by Fission-track (FT) analysis. The purpose of this article is to present apatite and zircon fission-track data, and U–Pb granite ages that provide information about the cooling histories of a rock which can be crucial in comprehending the exhumation episodes of the study area, in particular, and the region, in general. Granite samples were collected along the same vertical profile at different elevation, 178–944 m.a.s.l. These samples were used to determine Fission-Track and crystallization ages. HeFTy software was employed to interpret the cooling histories of the samples using forward and inverse models. The inverse model was an approach of reproducing the observed data, and it was carried out only for fission-track data from the apatite grains. And it was constructed after generating a number of forward models, where in each of these models the predicted apatite fission-track parameters were compared to the measured values. The apatite fission track (AFT) and zircon fission track (ZFT) data indicated expected age trends, *i.e.* the older ages at higher elevations and the younger ages at lower elevations. Similarly, the data shows that the apatite and zircon FT ages appear younger than the age of the rock crystallization. The U–Pb age in zircon consistently suggest the age of the granite is Late Triassic.

**Table:** Specifications table

Subject area	Geology
More specific subject area	Tectonics, Thermochronology
Type of data	Figure, graph, and table
How data was acquired	Five Samples (CH-6 to CH-10) were collected from the Main Range granite (Fig. 1). The elevation difference between Sample CH-6 and Sample CH-10 is 766 m; the first sample was collected at an altitude of 944 m above sea level (a.s.l.), and subsequently the other samples were collected from elevations of 717 m, 577 m, 340 m, and 178 m (a.s.l.). Among these samples, four of them (CH-6 to CH-9) were used to determine apatite fission-track age (FTA), whereas three of them (CH-6, CH-8, and CH-10) were used to determine zircon FTA.
Data format	Raw, analyzed
Experimental factors	Sample crushing, separation of grains, mounting, polishing, and etching for measurement of fission-track lengths and ages [1].
Experimental features	Thermal modeling, forward and inverse models were constructed to interpret the cooling histories of the samples and their exhumation through the upper crust.
Data source location	Main Range granite, east of the Kinta Valley, Cameron Highland, Peninsular Malaysia.
Data accessibility	Data is with this article.

**Table:** 

**Value of data** • The absolute age of the Main Range granite has been a subject of debate; nonetheless, the U–Pb age data provided in this article strengthens the earlier estimated age ranges indicated in a few published works. • The data will serve as additional input for researchers who will be working to understand the detailed exhumation history of the study area, in particular, and the region, in general. • The carbonate rocks adjoined to the granite are partially metamorphosed, and the data will give crucial information for researchers who will try to investigate the timing of contact metamorphism.

## 1 Data

The apatite fission track (AFT) and zircon fission track (ZFT) data indicate expected age trends, *i.e.* the older ages at higher elevations and the younger ages at lower elevations, except AFT age for sample CH-9 (Table 1). The biased older age in sample CH-9 is attributed to the presence of a single, slightly older grain in this sample, which is dominated by tiny and difficult to work with apatite grains. The statistical analysis indicates that all grain ages passed the chi-squared test (P(χ^2^) > 5%), which exhibits the homogeneity of the ages of the grains population, *i.e.* shows a high probability of coming from a single-age population.

**Table 1:** Summary of Apatite and Zircon fission track data.

Sample	Elev (m)	Mineral	Accepted Spots	N	Mean [U] (ppm)	Mean Dpar (μm)	Mean Dper (μm)	Pooled Age (Ma) ±95% CI	P (χ^2^) (%)
CH-6	944	Apatite	40	2049	137.9	2.02	0.34	33.93	89.2
								−2.11,+2.25	
		Zircon	30	2525	387	–	–	97.47	57
								−8.51,+9.32	
CH-7	717	Apatite	40	1459	82.9	1.92	0.36	30.98	88
								−2.08,+ 2.23	
CH-8	577	Apatite	31	86	26.8	1.89	0.36	7.46	86.7
								−1.67,+2.16	
		Zircon	4	321	524.8	–	–	79.26	7.5
								−15.9,+19.9	
CH-9	340	Apatite	13	34	30.1	1.82	0.36	16.54	64
								−4.94,+7.04	
CH-10	178	Zircon	4	303	481.2	–	–	68.7	5.3
								−15.5,+19.9	
								

Where, N = number of tracks; U = uranium content; Dpar = mean track etch pit diameter parallel to c-axis; Dper = mean track etch pit diameter perpendicular to c-axis.

After undertaking a number of test runs, many statistically good and acceptable fits were obtained, and among them the best fits were chosen. Fig. 2a–c indicate the results obtained in the inverse model, and the high goodness-of-fit (GOF) marks the most likely approximate value between the measured and the modeled age and fission-track length. The linear cooling episode experienced by the samples used in this study is shown in the inverse models, and here the apatite fission track ages indicate the time passed since the samples cooled below the closure temperature, *i.e.* ∼110–120 °C. These models clearly show the existence of two rapid cooling episodes after the Mid Eocene, with relatively slower cooling in between (Fig. 2a and b). Thus, the early episode of cooling brought the upper two samples, *i.e.* CH-6 and CH-7, into the cooler zone, fixing them in older ages. However, a later episode occurred during the Late Miocene and resulted in bringing the base of the sampled section into cool temperatures as experienced by the top samples.

**Fig. 1:**
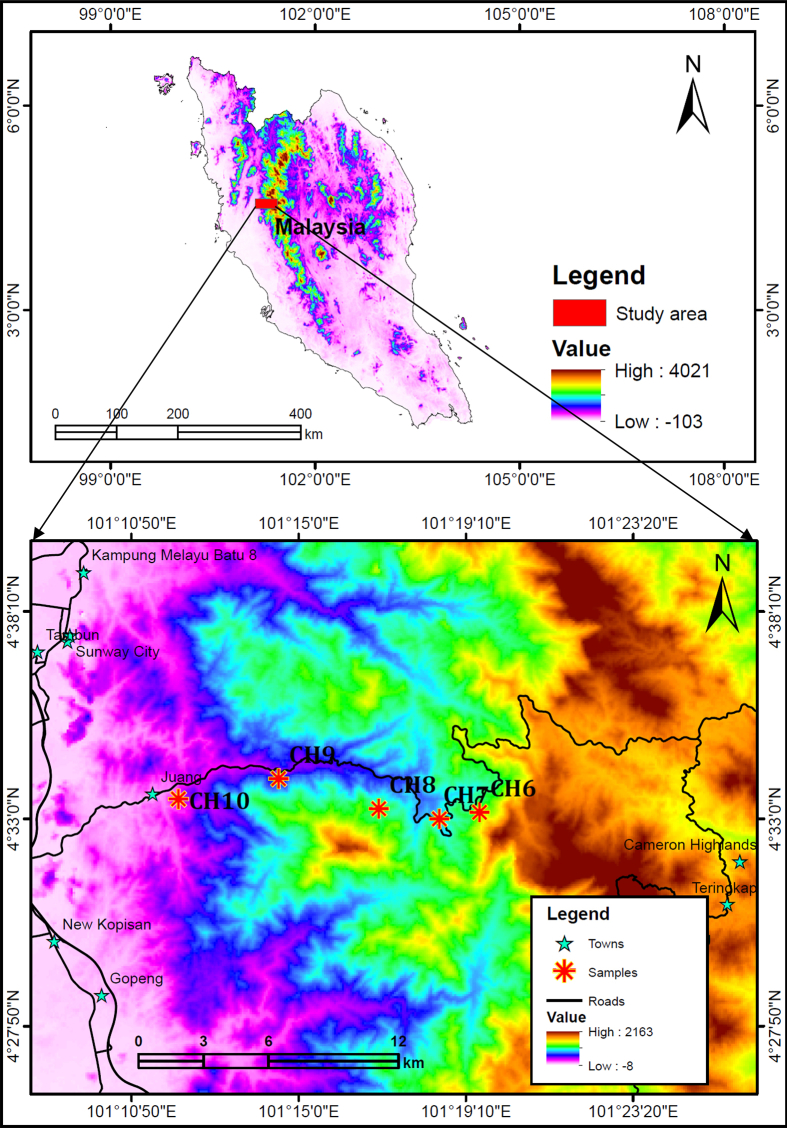
Location map of the study area.

**Fig. 2:**
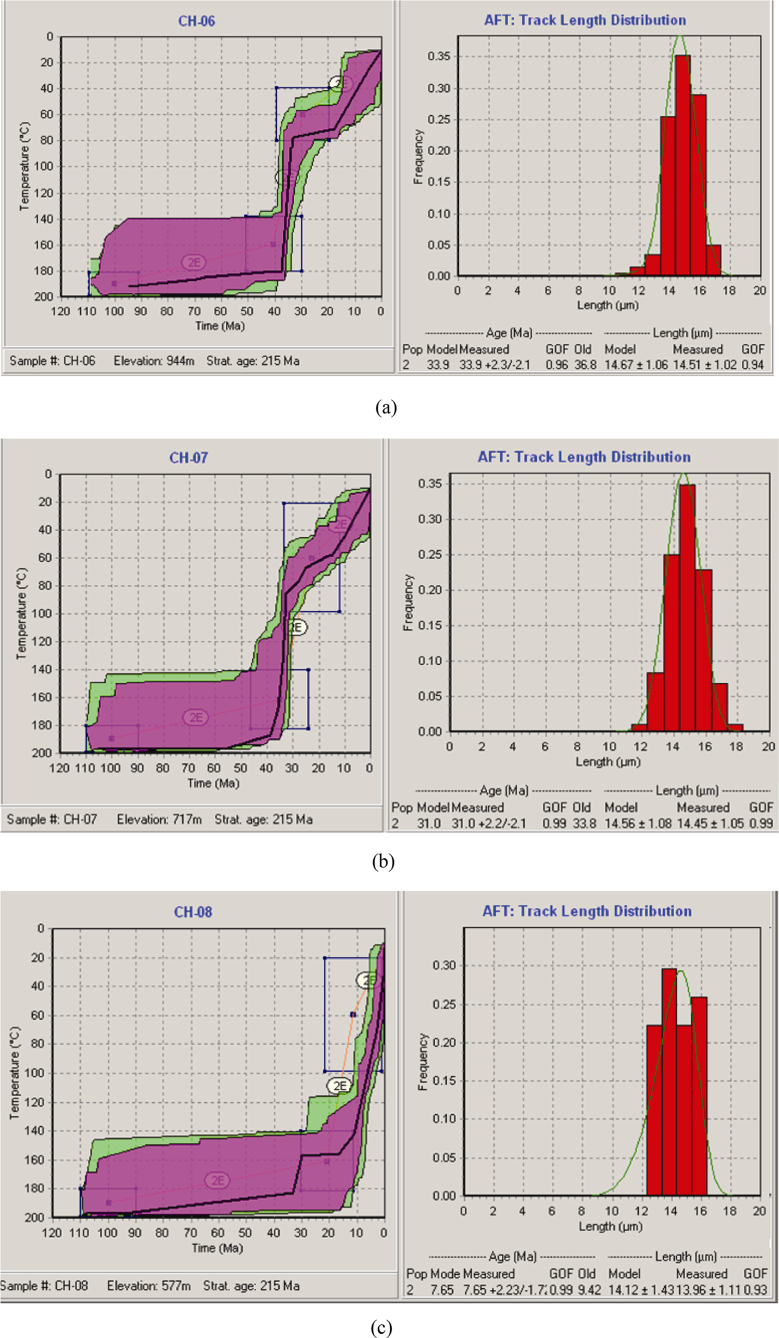
Inversion model of time-temperature history for Samples (a) CH-06, (b) CH-07, and (c) CH-08. Based on the montecarlo search method and 20, 000 time-temperature tested paths.

The inverse model, for Sample CH-6 (Fig. 2a), suggests the onset of fast cooling during the Upper Eocene, and continued up to the Early Oligocene. The second cooling event is marked by a relatively slower cooling which lasts up to the Early Miocene. Then, the last episode of fast cooling continued. Like Sample CH-6, Sample CH-7 (Fig. 2b) has also underwent similar cooling history, where the first episode of cooling is characterized by faster rate, which took place during Upper Eocene to Early Oligocene, and ensued by the later less faster episode. Although both samples have experienced faster cooling, the second younger episode in sample CH-7 is not as pronounced as CH-6, which could be as a consequence of less resistance to annealing by the latter sample (with measured track length, MTL = 14.51 ± 1.02 μm) than by the former one (with MTL = 14.45 ± 1.05 μm) (Fig. 2a and b). Sample CH-8 shows much more recent event of cooling (Fig. 2c). After the emplacement of the upper two samples in a cooler temperature zone, during the Late Eocene to Early Oligocene, the lower sample (CH-8) emplaced into the lower temperature zone during the Late Miocene.

**Fig. 3:**
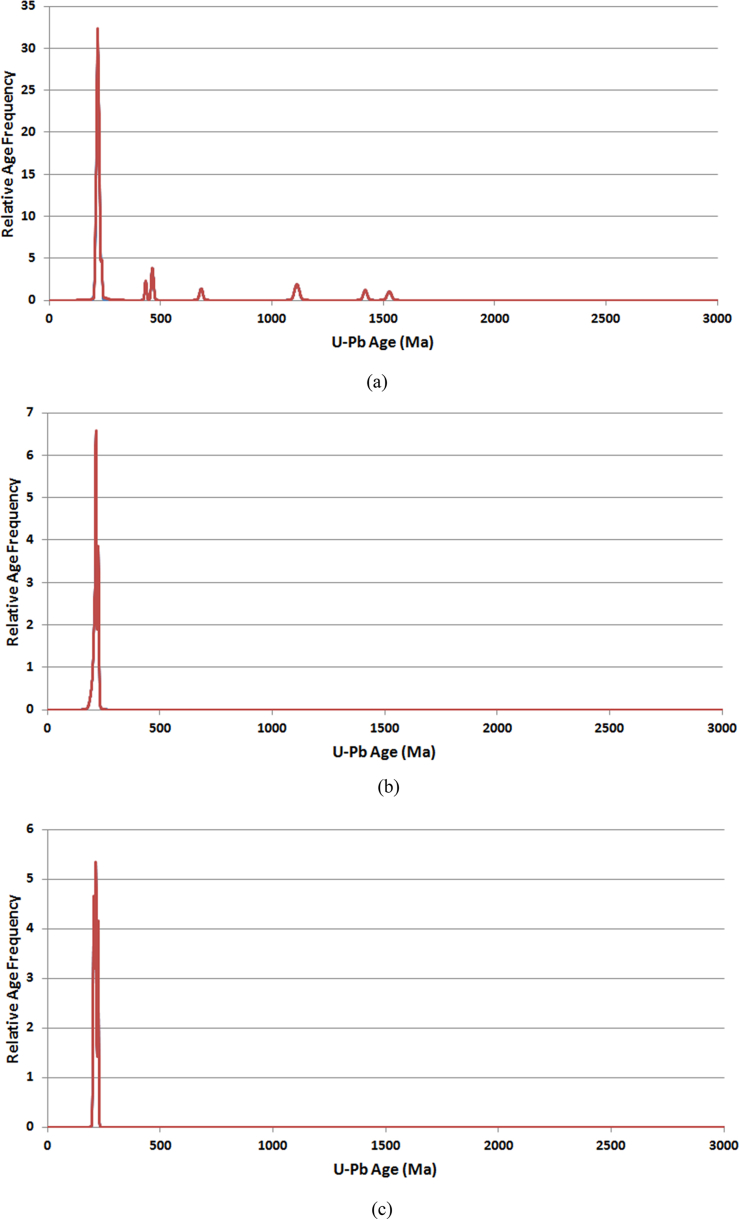
Graphs illustrating UPb age in zircon for the respective samples (a) CH 6, (b) CH 8 and (c) CH 10. The UPb age indicates the time of intrusion about 220 Ma.

**Fig. 4:**
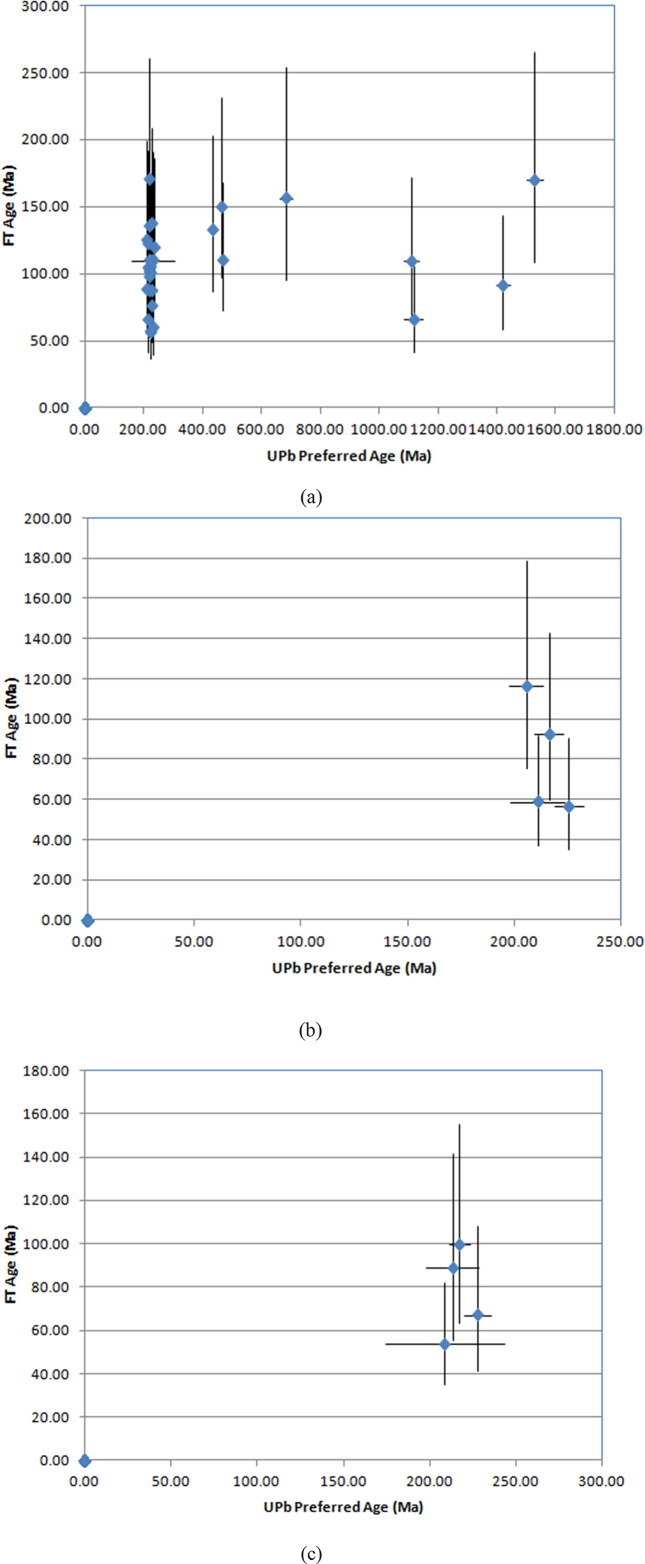
Graphs illustrating UPb age vs. FT age in zircon for the respective samples (a) CH 6, (b) CH 8 and (c) CH 10.

The apatite and zircon FT ages appear younger than the age of the rock crystallization. The U–Pb age in zircon (Fig. 3a–c) consistently suggest the age of the granite is in the Late Triassic, almost similar to previous estimates indicated in [2], [3]. Furthermore, Fig. 4a–c indicates U–Pb versus FT age in zircon for samples CH-6, CH-8 and CH-10.

## 2 Experimental design, materials and methods

### 2.1 Study area

The study area is located in the Main Range granite of Kinta Valley, western part of Peninsular Malaysia. The valley is bounded by granite ranges; in the west by the Klendang Range and in the east by the Main Range. It is characterized by remnant limestone hills, the rocks of which have been dated Silurian to Permian [4], [5], [6]. The limestone has been affected by variable degree of metamorphism. The impact of metamorphism is particularly strong at the contact zone with the granite and visible with the crystalline nature of the rock. However, the extent of metamorphism appears to be diminishing away from the granite.

The Main Range Granite, which adjoins the Kinta Valley carbonate successions and forms the backbone watershed mountain range of the Peninsula, is the result of several large granitic batholiths [7]. This plutonic rock was emplaced by the activity related to the Late Triassic uplift from plate boundary stresses along the western edge of the Malay Peninsula [8]. The absolute age of the Main Range granite has been a subject of debate; however, summary of most published data sets indicated that the Main Range granitoids are about 200–230 Ma old [2], [9], [10], [11].

### 2.2 Sample collection

Five Samples (CH-6 to CH-10) were collected from the Main Range granite at the foot of the Cameron Highland (Fig. 1). The elevation difference between Sample CH-6 and Sample CH-10 is 766 m; the first sample was collected at an altitude of 944 m above sea level (a.s.l.), and subsequently the other samples were collected from elevations of 717 m, 577 m, 340 m, and 178 m (a.s.l.). Since the samples were collected almost along the same vertical profile, they are considered to have passed through similar path of thermal conditions. Among these samples, four of them (CH-6 to CH-9) were used to determine apatite fission-track age (FTA), whereas three of them (CH-6, CH-8, and CH-10) were used for zircon FTA.

The granite samples used in this study were prepared and analyzed by A to Z, Inc., USA. The reader is referred to [1] for the details of the techniques, which ranges from sample crushing, separation of grains, mounting, polishing, and etching for measurement of fission-track lengths and ages. After the fission-tracks data were obtained, thermal models were constructed for apatite fission-tracks.

### 2.3 Thermal modelling

In order to interpret the cooling histories of the samples and their exhumation through the upper crust, forward and inverse models were constructed. Commonly, forward models are performed to predict what fission-track age and track length distribution one gets for a particular time-temperature history. Thus, HeFTy software was employed to produce those models [12], [13]. By consistently varying the time-temperature path, the software updates the predicted fission-track parameters. This simulation, in turn, was used to interpret the real data from laboratory measurements. Secondly, inverse modeling was performed. This model is an approach of reproducing the observed data, and it was carried out only for fission-track data from the apatite grains. It was constructed after generating a number of forward models, where in each of these models the predicted apatite fission-track parameters were compared to the measured values. And then the good forward models were used to interpret the time-temperature histories. In these inverse models, the default Monte Carlo modeling scheme (search method) was employed. In order to get a good fit or solution, many time-temperature paths can be tested, and for this study 20,000 paths were tested for each model. Some constraints including age for the earliest intrusion, maximum annealing temperature, and surface temperature were placed; thus, it is supposed that the HeFTy program (i) will have the liberty to determine the possible time range in which the sample entered to a certain temperature range where the tracks are retained, and (ii) will search many possible paths (out of the 20,000 paths), and will find any likely cooling phenomenon and associated timing. And this enabled us to determine which of the random paths have passed certain statistical criteria, so as to exhibit the acceptable or good fit to the data.
